# Mitogen-activated protein kinase signaling causes malignant melanoma cells to differentially alter extracellular matrix biosynthesis to promote cell survival

**DOI:** 10.1186/s12885-016-2211-7

**Published:** 2016-03-05

**Authors:** Anna Afasizheva, Alexus Devine, Heather Tillman, King Leung Fung, Wilfred D. Vieira, Benjamin H. Blehm, Yorihisa Kotobuki, Ben Busby, Emily I. Chen, Kandice Tanner

**Affiliations:** Laboratory of Cell Biology, Center for Cancer Research, National Cancer Institute, National Institutes of Health, 37 Convent Dr., Bethesda, MD 20892 USA; Laboratories of Genitourinary Cancer Pathogenesis, Center for Cancer Research, National Cancer Institute, National Institutes of Health, 37 Convent Dr., Bethesda, 20892 MD USA; National Centers for Biotechnology Information, National Library of Medicine, National Institutes of Health, Bethesda, 20892 MD USA; Proteomics Shared Resource at the Herbert Irving Comprehensive Cancer Center & Department of Pharmacology, Columbia University Medical Center, New York, 10032 NY USA

**Keywords:** Melanoma, Fibronectin, Extracellular matrix, MAPK, Integrin, Tenascin-C, Vemurafenib, Dabrafenib

## Abstract

**Background:**

Intrinsic and acquired resistance to drug therapies remains a challenge for malignant melanoma patients. Intratumoral heterogeneities within the tumor microenvironment contribute additional complexity to the determinants of drug efficacy and acquired resistance.

**Methods:**

We use 3D biomimetic platforms to understand dynamics in extracellular matrix (ECM) biogenesis following pharmaceutical intervention against mitogen-activated protein kinases (MAPK) signaling. We further determined temporal evolution of secreted ECM components by isogenic melanoma cell clones.

**Results:**

We found that the cell clones differentially secrete and assemble a myriad of ECM molecules into dense fibrillar and globular networks. We show that cells can modulate their ECM biosynthesis in response to external insults. Fibronectin (FN) is one of the key architectural components, modulating the efficacy of a broad spectrum of drug therapies. Stable cell lines engineered to secrete minimal levels of FN showed a concomitant increase in secretion of Tenascin-C and became sensitive to BRAF^V600E^ and ERK inhibition as clonally- derived 3D tumor aggregates. These cells failed to assemble exogenous FN despite maintaining the integrin machinery to facilitate cell- ECM cross-talk. We determined that only clones that increased FN production via p38 MAPK and β1 integrin survived drug treatment.

**Conclusions:**

These data suggest that tumor cells engineer drug resistance by altering their ECM biosynthesis. Therefore, drug treatment may induce ECM biosynthesis, contributing to *de novo* resistance.

**Electronic supplementary material:**

The online version of this article (doi:10.1186/s12885-016-2211-7) contains supplementary material, which is available to authorized users.

## Background

Molecular studies have determined that aberrant signaling in the mitogen-activated protein kinase (MAPK) signaling cascade (Raf-MEK1/2-ERK1/2) is a crucial step in melanocytic neoplasia, ultimately deregulating the activity of key transcription factors that control cell growth, differentiation, and survival [1]. Specifically, gain of function mutations in which the 600 valine residue of the BRAF kinase is substituted with glutamic acid (BRAF^V600E^) are present in ~40 % of patients presenting with malignant melanoma [2]. Survival is enhanced if detection, treatment and surgical excision occur when the tumor burden is restricted to a primary site; however, once the disease has metastasized to distant organs such as the lung, brain, bone, and liver, prognosis is poor, with a median survival of ~ 9 months [3]. These dismal statistics are in part due to the intrinsic resistance of melanoma to platinum-based chemotherapeutics and its rapid acquisition of resistance to initial therapeutic interventions [4, 5]. Present treatment strategies involve small molecule inhibitors of this mutant BRAF kinase, such as vemurafenib and dabrafenib [6, 7]. However, these therapies are only effective in a subset of patients: those that harbor the more prevalent BRAF^V600E^ and the less common BRAF^V600K^ mutations [8]. While drugs targeting this specific isoform are among the most promising melanoma treatments, patients eventually relapse due to reactivation of the MAPK signaling cascade, specifically ERK activity [5, 9]. To overcome *de novo* resistance, a combinatorial treatment of vemurafenib with a MEK inhibitor is administered in an effort to combat reactivation of the MAPK pathway [10–12]. However, mechanisms that underlie acquired resistance after treatment with multiple inhibitors of this pathway remain elusive.

Drug resistance has been shown to be mediated by tissue architecture and cell-adhesion [13, 14]. In particular, cell adhesion-mediated drug resistance (CAM-DR) is an emergent phenotype associated with cell-cell adhesion or 2D adhesion to extracellular matrices. Myeloma cells cultured as monolayers that had adhered to fibronectin were resistant via upregulation of α4 β1 integrin compared to cells treated in suspension [15]. Similarly, tumor cells grown as spheroids show increased resistance to therapy compared to the same cells that are dissociated and grown as monolayers [16]. However, the observed acquired drug resistance following multiple targeting of the MAPK pathway is not readily explained by CAM-DR [12]. Because this reactivation attenuates drug response, it may also contribute to the development of acquired resistance [12].

The tumor microenvironment is emerging as a critical factor in malignant progression, metastasis and tumor etiology [17, 18]. To explore mechanisms that drive tumors to overcome and survive under unfavorable conditions, we aimed to delineate tumor-induced microenvironmental responses to the stress induced by drug therapeutics. Tumor cells actively modulate the host environment by secreting cytokines that reprogram stromal cells to change the extracellular matrix (ECM) milieu, thus creating a *de novo* microenvironment [17, 18]. While immunotherapy and monoclonal antibodies targeting tumor angiogenesis have shown promising results, many microenvironmental targets remain underexplored [18]. For example, overexpression of secreted ECM proteins such as fibronectin (FN) has been found in several solid carcinomas, and postulated to be beneficial for tumor growth and instrumental in the establishment of an ideal microenvironment [19]. Furthermore, heterogeneous expression of ECM components within tumors has been observed [20]. Pathologists have long associated the presence of abundant ECM proteins in tumors with poor prognosis and an expected dismal response to therapeutic intervention [21].

Recently, a study showed that non-small cell lung cancer cells induced FN biogenesis via p38 MAPK in response to treatment with cetuximab (targeting the EGF receptor upstream of the MAPK signaling pathway) [22]. This response was found to blunt the cytotoxic effects of cetuximab and reduced sensitivity to radiotherapy in in vitro and in vivo murine models. FN biogenesis may also reduce the efficacy of drugs targeting the BRAF kinase. Earlier observations found that a cocoon of ECM proteins, including FN, laminin, collagen IV and Tenascin C, protect small lung cancer cells from chemotherapy-induced apoptosis [23]. We hypothesized that melanoma cells modulate secretion of not only FN, but also other ECM molecules to survive drug treatment. An important question is whether baseline ECM expression per se can predict cell survival and drug resistance. Furthermore, is upregulation of ECM proteins a reaction to evolutionary pressure following drug treatment, the result of selection for pre-existing resistant subpopulations, or a combination of both? Seeking to identify and determine the temporal regulation of the secreted ECM proteins, we focused on two isogenic cell lines to mimic intratumoral heterogeneity. Our results indicate that tumor cells adjust their 3D microenvironment by modulating secretion of FN and Tenascin-C (TNC), thereby blunting the effects of MAPK pathway inhibition. We show that only clones that can modulate their ECM secretion in response to pharmaceutical stress survive. Mechanistically, FN biogenesis via p38 MAP kinase via β1 integrin is induced following pharmaceutical treatment of the MAPK signaling pathway. Also, our data suggests that biogenesis of TNC is an possible alternative to impaired FN production. This study indicates a critical role for *de novo* ECM biogenesis as a mechanism driving acquired resistance to drugs targeting MAPK signaling in cutaneous melanoma.

## Methods

Human melanoma cell lines, A375 (cat. No. CRL-1619) and A375.S2 (cat. No. CRL-1872) cells were obtained from American Type Culture Collection (ATCC, Manassas, VA) and cultured on tissue culture flasks as described by the ATCC. A375 and A375.S2 cells were grown as monolayers in DMEM high glucose media (Life Technologies, Carlsbad, CA) supplemented with final volume of 10 % Fetal Bovine Serum (FBS), 1 % MEM non-essential amino acids, L-Glutamine, Penicillin and Streptomycin. Cells cultured with the media was refreshed every 2-3 days.

### Animal studies

Animal studies were conducted under protocols approved by the National Cancer Institute, and the National Institutes of Health Animal Care and Use Committee. Briefly, 5 × 10^5^ A375 cells were injected via tail vein into 8–10-week old female nu/nu mice. Three weeks after injection mice were euthanized and lungs were extracted and inflated and fixed in 4 % paraformaldehyde solution for 12 h and then prepared for histological staining. Lungs were then embedded in paraffin prior to sectioning on a microtome. Following antigen retrieval using xylene, samples were rehydrated and serial sections; 8 μm thick were labeled for specific stains of human fibronectin and DAPI.

### Human tissue arrays and RNAseq

Human tissue arrays (cat # T386) were purchased from US Biomax (US Biomax, Rockville, MD) where tissue biopsies of human normal skin, melanoma with and without metastasis were examined by immunofluorescence. Briefly, antigen retrieval using xylene as described for mouse tissue was conducted and following re-hydration. Samples were stained for human Fibronectin and DAPI. Public RNAseq datasets were submitted to National Center for Biotechnology Information (NCBI) and examined for expression of extracellular matrix proteins [24] and a search was done for public studies that involved ECM in melanoma (http://www.ncbi.nlm.nih.gov/pubmed/?term=tenascin%20C%20AND%20melanoma). Briefly, normal melanocyte cell lines were compared to melanoma cell lines. In addition, data before and after treatment in human patients were also mined from publicly available databases.

### 3D cell culture

Cells were seeded at a concentration of 2 × 10^5^ cells per ml of Matrigel (BD Biosciences, San Jose, CA), which polymerized to form a hydrogel at 37 °C. Media was added to the hydrogels after 30 min to ensure complete gelation. Cells were harvested after growth in 2D and embedded in laminin-rich extra cellular matrix (lrECM) (BD Biosciences) at 37 °C, in 5 % CO_2_ as previously described [25]. Clonally derived multicellular aggregates were examined 10 days later for morphology and protein analysis.

### Conditioned media collection and protein preparation

For 2D culture, cells were seeded at 5 × 10^5^ cells per well in a six well plate with regular media. After 24 h the regular media was removed and cells were washed twice with PBS, and serum-free media was added. Conditioned (serum-free) media and cell lysate were collected after 24 h. For 3D culture, cell washing and refresh with conditioned medium was conducted repeated at day 8, following sample collection at day 9. For 3D cultures, cells were also harvested from lrECM for immunoprecipitation as previously described [25]. Melanoma cells and normal human melanocytes were lysed in Mammalian Protein Extraction Reagent (Thermo Scientific, Waltham, MA), 1× protease inhibitor cocktail (Calbiochem, Billerica, MA) and 1× phosphatase inhibitor cocktail 2 & 3 (Sigma-Aldrich, St. Louis, MO) followed by centrifugation (13,200 rpm, 4 °C, 5 min), after which the supernatants were stored at -80 °C until use. Protein concentrations were determined with the Pierce^®^ BCA Protein Assay kit (Thermo Scientific), using BSA as the concentration standard. Extracted proteins were then resolved using 4–12 % Bis-Tris Gels (Life Technologies) and subsequently transferred to PVDF membranes (Life Technologies) for immunoblotting.

### Western blot analysis

PVDF membranes were washed and blocked with 1 % BSA in TBS (20 mM Tris-HCl, 150 mM NaCl, pH 7.4) containing 0.1 % Tween-20 (TBS-T) and incubated with the primary antibodies. Next, the membranes were incubated with horseradish peroxidase-conjugated donkey anti-rabbit IgG (GE Healthcare, Buckinghamshire, UK) or sheep anti-mouse IgG (GE Healthcare). Finally, the immunoblot was incubated with an enhanced chemiluminescence (ECL) reaction system (Invitrogen) and the ECL signals were visualized by film. The antibodies were used at the following dilutions: anti-FN (1:1,000), anti-αv integrin (1:1,000), anti-β1 integrin (1:1,000), anti-phospho-p44/42MAPK (1:1,000), anti-p44/42MAPK (1:1,000), and anti-GAPDH (1:2,000). Except anti-FN (Origene, Rockville, MD), antibodies are purchased from Cell Signaling Technology (Danvers, MA).

### RNA extraction, reverse transcription, and RT-PCR

Total RNA was extracted using an RNeasy mini Kit (Qiagen, Valencia, CA) and first-strand complementary DNAs (cDNAs) were synthesized from 500 ng of each total RNA preparation with the Quantitect Reverse Transcription kit (Qiagen) according to the manufacturers’ instructions. The PCR products were detected by SYBR Safe DNA gel stain (Invitrogen) after separation in 1 % agarose gel. β-actin was used as a housekeeping gene to evaluate and compare the quality of different cDNA samples. The primer sequences and the expected sizes of PCR products were as follows: Human FN 1, forward, 5’-ACAGGAAAGAGATGCGCCAA-3’, reverse, 5’-GGAAGAGTTTAGCGGGGTCC-3’ (403 bp); Human β-actin, forward, 5’-AGCCTCGCCTTTGCCGA-3’, reverse, 5’-CTGGTGCCTGGGGCG-3’ (174 bp). AmpliTaq Gold with GeneAmp and dNTP Mix (Applied Biosystems, Branchburg, NJ) were used for the RT-PCR analysis. We used a denaturing step of 94 °C for 5 min, followed by a cycling program of 94 °C for 10 s, 58 °C (Human FN1) or 60 °C (Human β-actin) for 60 s, and 72 °C for 60 s for 40 cycles, and a final holding stage at 4 °C.

### Materials for mass spectroscopy

Dithiothreitol (DTT), acetonitrile (ACN), ammonium bicarbonate, trifluoretic acids (TFA), and iodoacetamide (IAA) were from Thermo Fisher Scientific. Trypsin Gold, mass spectrometry grade, was from Promega (Madison, WI). Nanopure water was prepared with use of Milli-Q water purification system (Millipore, Billerica, MA).

### Preparation for in-gel protein digestion

For MS analysis, cells were grown in 1 % dialyzed FBS in 10 cm dishes and conditioned media (CM) collected every 2 days; protease and proteinase inhibitors were added immediately*.* CM was centrifuged at 3,500 rpm for 30 min to remove cell debris and the supernatant was concentrated using Amicon Ultra-15, Ultracel-3 K (ThermoFisher, Cat No: UFC900324) following the manufacturer’s instructions until the volume was reduced to ~300ul. Protein concentration was determined using BCA assay. 30 μg of proteins were separated using precast 4–20 % Tris-Glycine SDS-PAGE gels (Life Technologies). Total protein resolved in the polyacrylamide gel was stained with Coomassie blue (Sigma). The gel was destained, reduced, alkylated, and dried for in-gel digestion. Biological replicates at each time point (*n* = 3) were run on the same gel and processed simultaneously.

### In-gel protein digestion

The dried gel pieces were rehydrated and digested in 80 μL of 12.5 ng/μL Trypsin Gold/50 mM ammonium bicarbonate at 37 °C overnight. After the digestion was complete, condensed evaporated water was collected from tube walls by 5 s centrifugation using a benchtop microcentrifuge (Eppendorf, Hauppauge, NY). The gel pieces and digestion reaction were mixed with 50 μL 2.5 % TFA and rigorously mixed for 15 min. The solution with extracted peptides was transferred into a fresh tube. The remaining peptides were extracted with 80 μL 70 % ACN/5 % TFA mixture using rigorous mixing for 15 min. The extracts were pooled and dried to completion (1.5–2 h) in SpeedVac. The dried peptides were reconstituted in 30 μL 0.1 % TFA by mixing for 5 min and stored in ice or at −20 °C prior analysis.

### LC-MS/MS analysis

Samples were centrifuged at 14,000–16,000 × g for 10 min to remove particulate material. Five microliters of each sample were injected into a self-packed fused silica nano column, which had been pulled to a 5 μm i.d. tip using a P-2000 CO_2_ laser puller (Sutter Instruments, Novato, CA), then packed with 13 cm of 3 μm C18 reverse phase (RP) particles (Magic *C18* AQ 3 μm (Michrom. Bioresources, Auburn, CA)) and equilibrated in 5 % acetonitrile, 0.1 % formic acid. Full MS spectra were recorded on the peptides over a 400 to 2000 m/z range by the Orbitrap, followed by five tandem mass (MS/MS) events sequentially generated by LTQ in a data-dependent manner on the first, second, third, and fourth most intense ions selected from the full MS spectrum (at 35 % collision energy). Mass spectrometer scan functions and HPLC solvent gradients were controlled by the Xcalibur data system (Thermo Finnigan, San Jose, CA).

### Database search and interpretation of MS/MS datasets

Tandem mass spectra were searched against a concatenated target-decoy database containing the forward and reverse sequences of the target database using the Proteome Discoverer 1.4 and SEQUEST search engine (Thermo Finnigan). The target sequence was downloaded from a human Uniprot database (database released on November 13, 2013) and 124 common contaminant proteins. The search algorithm was configured to specify the following parameters: precursor tolerance, 10 ppm; fragment tolerance, 0.5 Da; static modification, cysteine carbamidomethylation; and fully tryptic status. SEQUEST search results from Proteome Discoverer were further analyzed by Scaffold (Proteome Software Inc., Portland, OR). The discriminant score was set such that a false positive rate of 1 % was determined based on the number of accepted decoy database peptides. Scaffold outputs label-free quantification based on precursor ion intensity for data from Proteome Discoverer. Precursor intensity refers to the area under an MS1 spectrum peak corresponding to a specific peptide. We used normalized total precursor intensity to derive quantitative values in this paper. Statistical analysis was performed by Qlucore Omics Explorer (Qlucore AB, Lund, Sweden). Two group comparisons (a paired *t*-test) was used to compare the mean value of relative abundance based on normalized total precursor intensity from three biological replicates for each protein identified in conditioned media collected in day 2 and day 10. The heatmap was generated based on hierarchical clustering of global protein expression filtered by the *p*-value (*p* < 0.05).

### Cell growth inhibition assay by MTT method

Cellular toxicity to drugs was measured by the MTT (3-(4,5-Dimethylthiazol-2-yl)-2,5-diphenyltetrazolium) method or CCK-8 (Dojindo Molecular Technologies, Inc., Rockville, MD) method following manufacturer’s instructions.

### Generation of shFN1 and shFN2 stable cell lines

Plasmids encoding FN knockdown shRNAs were isolated from Mission shRNA Bacteria Glycerol Stocks NM_002026/TRCN0000293839 and NM_002026/TRCN0000293840 (designated shFN1 and shFN2, respectively). MISSION^®^ pLKO.1-puro Non-Mammalian shRNA control plasmid (SCH002, Sigma) was amplified in MAX Efficiency^®^ DH5α™ Competent Cells (Invitrogen) for 16 h at 30 ^o^C. Plasmids were extracted from ampicillin-supplemented LB liquid cultures amplified from single colonies over 16 h at 30 ^o^C using Qiagen Plasmid Midi Kit. Individual plasmids were co-transfected with lentiviral packaging plasmids (Invitrogen) into 293FT cells by Fugene6 Transfection Reagent (Roche, Basel, Switzerland). Viral supernatant was harvested after 48 and 72 h and concentrated by ultracentrifugation at 26,000 rpm through a 20 % sucrose gradient.

### Lentiviral transduction

Melanoma cells were transduced with virus using ExpressMag Transduction System (Sigma). Briefly, viral supernatant was incubated with magnetic beads at room temperature for 15 min, added to the adherent cells and placed on a strong magnetic plate at 37 ^o^C for 12 min. Media was replenished 16 h later and the viral mixture disposed in compliance with NIH policy. Transduced cells were selected with 2 μg/ml puromyocin (InvivoGen, San Diego, CA).

### Immunofluorescence

For examination of 2D culture, cells were seeded directly onto glass chambers with a coverslip-bottomed no. 1.0, (Nalge, Nunc) according to previously described methods [25]. For 3D interrogation, samples were prepared as previously described [25]. Samples were rinsed twice with PBS, fixed with 4 % paraformaldehyde solution for 20 min, permeabilized with 0.5 % Triton X-100 for 20 min and rinsed and blocked in 5 % horse serum for 1 h at room temperature. Cells were then incubated with anti-FN1 antibody (1:100) and anti-actin antibody (1:100) for 12 h at 4 °C in 5 % horse serum. After incubation with primary antibody, cells were rinsed three times with PBS and then incubated with secondary antibodies (1:200) for 2 h at 4 °C. Cell nuclei were stained with DAPI (Sigma). Images were acquired with an upright Zeiss LSM 780 Meta confocal microscope (Zeiss, Jena, Germany).

### Imaging specifications

One-photon, confocal, 2-dimensional images of 512 × 512 pixels (lateral dimensions) were acquired with a 1.4 NA 40 × oil-immersion objective. We sequentially excited our sample with the 405 nm, 488 nm lines from an argon ion laser with a power of < 3 % (total power 30 mW) and 546 nm from a solid-state laser (power of < 10 %). A secondary dichroic mirror, SDM 560, was employed in the emission pathway to delineate the red (band-pass filters 560–575 nm) and green (band-pass filters 505–525 nm) and blue channels (480-495 nm) at a gain of 400 on the amplifier. The laser power for the 543 nm setting was set at < 3 % of the maximum power and the gain on the detectors was set to 450.

### 3D soft agar assay

Anchorage-independent proliferation was quantified by soft agar assay. Cells were suspended in 1 mL of 0.36 % (w/v) bacto-agar (BD Biosciences) overlaid by a 0.6 % (w/v) bacto-agar support in 6-well plates in triplicates at densities of 5 × 10^3^ cells per well for A375, A375.S2, A375Scr, A375 shFN2, A375.S2 Scr, A375.S2 shFN2 lines, respectively. Plates were incubated at 37 °C in normoxic conditions. Pathway inhibitors were used at the following concentrations: 3.0 μM cisplatin (DNA-crosslinking agent), 3.0 μM VX-11e (ERK inhibitor), 3.0 μM vemurafenib (BRAF^V600E^ inhibitor) (Chemietek, Indianapolis, IN), and 50 nM paclitaxel (microtubule stabilizing agent) (Life Technologies). Pathway inhibitors were administered at the beginning of the experiment or initiated 14 days later. At the end of the experiment, wells were stained with 0.5 μg/mL of nitrotetrazolium blue chloride (Sigma-Aldrich). Colonies with > 20 μm in diameter were counted using the Bioreader Software and a BioSys BioCount 4000 Pro machine. Clonogenic survival for each line and condition was plotted as frequency of colony size normalized to a gaussian distribution. Experiments were performed in triplicate.

## Results

### Distinct temporal regulation of extracellular matrix protein production in response to drug treatment is dynamic

**Fig. 1:**
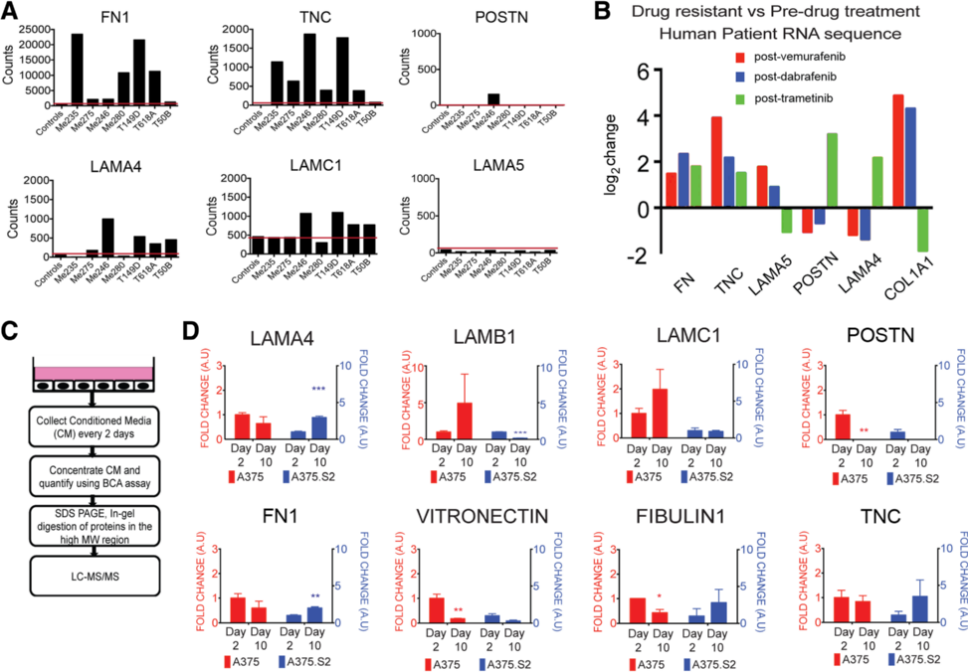
Distinct temporal regulation of ECM in response to drug treatment and in vitro culture conditions. **a** RNA sequence data show that FN is upregulated in melanoma cell lines and patients who have become resistant as a result of multiple drug treatments. Bar charts showing RNA sequence counts for control melanocytes compared to seven melanoma cell lines treated with BRAF inhibitor, where the red line shows the basal level of controls. **b** RNAseq-based expression levels for patients that were biopsied pre- and post-vemurafenib (*red*), dabrafenib (*blue*), and trametinib (*green*) treatment. **c** Schematics of experimental protocol using MS to identify secretomes of the isogenic human melanoma clones. **d** Dynamics of secreted ECM components show that temporal evolution of tumor biofilm differs as a function of tumor heterogeneity in cell culture. Quantitative comparison of ECM proteins in conditioned media reveals temporal differences in the amount of secreted proteins, highlighting the heterogeneity of baseline ECM biogenesis. * indicates a *p* value < 0.05, ** indicates a *p* value < 0.01 and *** indicates a *p* value < 0.001

Public RNAseq datasets submitted to National Center for Biotechnology Information (NCBI) were examined for expression of extracellular matrix proteins. Six melanoma cell lines consistently showed higher expression of FN1 (fibronectin) than pooled human melanocyte controls (Fig. 1a) [24]. The only other known ECM-related transcript consistently upregulated at the RNA level was TNC (Tenascin-C), which has been shown to be involved in melanoma progression [26]. Moreover, when a separate dataset was examined for RNAseq-based expression levels in human patients, pre- and post-vemurafenib (first column), dabrafenib (second column) and trametinib (third column) treatment, the same pattern of expression (presented as log_2_ (control)) was observed, with FN1 and TNC, but not other ECM-related transcripts, consistently upregulated at the RNA level [27] (Fig. 1b). This may occur in response to treatment, or due to selective survival of tumor cells that originally expressed higher levels of the ECM.

To determine the dynamic contributions made by tumor cells to the establishment of a *de novo* tumor microenvironment, we selected A375 and its derivative clone A375.S2, which are isogenic human melanoma cell lines that reflect established intratumoral heterogeneity. These cell lines harbor a BRAF^V600E^ mutation and are tumorigenic when injected into immune-compromised mice. Using a tandem mass spectrometry-based technique (LC-MS/MS), we analyzed the culture media conditioned by the parent and its derivative cell lines over the course of 10 days in 2D culture (Fig. 1c). We identified many ECM-related proteins were commonly or differentially secreted**.** The amount of these secreted ECM components was modulated as a function of time, thus illustrating the dynamics of tumor biofilm formation, and baseline secretion was also consistently distinct between the clones (Fig. 1d). Secretion of fibronectin (FN), in particular, varied significantly in amount and timing in A375 and A375.S2 cell lines. We thus reasoned that using the 3D biomimetic platforms, we could further address the heterogeneity of ECM secretion and dynamics, particularly as it relates to acquired resistance.

### FN deposition is increased in metastatic melanoma cells

**Fig. 2:**
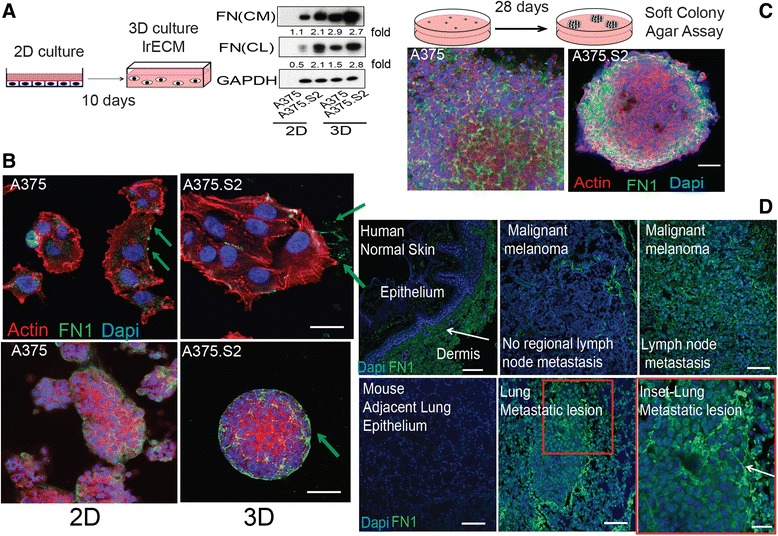
Fibronectin is a key architectural component in tumor biofilm in vitro and in vivo. **a** Top panel- Schematic of 3D sample preparation where cells were embedded in laminin-rich ECM (lrECM) and cultured for 10 days to recapitulate growth of melanoma with a basement membrane. Bottom panel- Immunoblots showing an increase in the levels of FN in conditioned media (CM) and cell lysates (CL) when cells are cultured in 2D vs. 3D. The A375 clone shows a threefold increase in FN production, whereas the A375.S2 clone shows a modest increase. Numbers next to the immunoblots represent fold of increase in fibronectin expression. **b** Micrographs showing the differences in assembly of secreted FN (*green*) between A375 and A375.S2 in both 2D and 3D culture. Green arrows show the locations of FN. Scale bars for 2D represent 5 μm and for 3D, 20 μm, respectively. **c** Schematic of 3D sample preparation for anchorage-independent growth where cells were embedded in soft agar and cultured for 28 days. Micrographs show that all FN is tumor-generated in this assay. Scale bars represent 50 μm. **d** Top panel- micrographs of human tissue arrays of normal skin as well as malignant and metastatic melanoma show that there is an increase in FN deposition within the tumor mass as a function of increasing metastatic potential, whereas FN deposition is restricted to the dermis in normal skin epithelium. Micrographs show increased FN deposition within the metastatic lung lesion compared to normal adjacent lung epithelium in the mouse following tail vein injection of melanoma cells. Scale bars represent 50 μm and 20 μm for the inset

Immunoblots of proteins extracted from cell lysates (CL) and conditioned media (CM) show that the A375 cells secreted less FN than the A375.S2 cells when they were cultured in 2D environment (Fig. 2a). We confirmed these cell lines express FN1 via RT-PCR (Additional file 1: Figure S1b). When these cell lines were cultured in laminin-rich ECM (lrECM), a complex matrix more closely mimicking in vivo conditions, both cell lines secreted more FN than in 2D culture (Fig. 2a). The A375 cells formed large, irregular “grape-like” aggregates, whereas A375.S2 clone formed smaller compact “masses” (Fig. 2b). The mean cross-section area and circularity of the A375.S2 colonies were significantly different from the A375 colonies (Additional file 1: Figure S1C). These differences in morphology and shape were similar to phenotypes observed for 3D culture of breast tumor cells [25]. Additionally, confocal immunofluorescence revealed that cells secreted FN and assembled FN-globular networks in 3D lrECM culture, in contrast to the fibrils observed in 2D culture (Fig. 2b). The immunofluorescence of colonies grown in soft agar further confirms that both clones can assemble FN structures in a foreign microenvironment, and that this FN is tumor-derived (Fig. 2c). To establish the relevance to human disease progression, we determined that FN levels vary as a function of malignancy in human tissue arrays. Compared to normal skin and non-metastatic melanoma, we observed increased FN deposition at sites of melanoma metastasis (Fig. 2d). FN deposition was specifically localized to the metastatic lesions rather than adjacent normal lung or lymph epithelium**.**

### Difference in tumor architecture affects MAPK inhibitor sensitivity

**Fig. 3:**
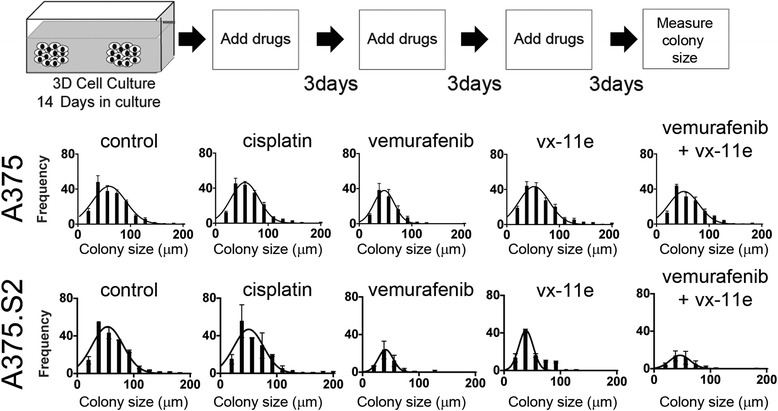
A375 and A375.S2 colonies show a differential response to drug treatment. Schematic showing the timing of drug administration for cells embedded in 3D soft agar with treatment commencing after colony formation. Growth of isogenic colonies in 3D soft-agar assays shows differential response to drug treatment. A375 colonies (*Top panel*) are largely insensitive to drug treatment, whereas A375.S2 (*Bottom panel*) cells form fewer colonies of smaller size as determined by diameter

Tumor architecture has been shown to be an important factor for determining drug responses in breast tumors cultured in 3D lrECM compared to the tissue culture [28]. To determine whether the efficacy of drugs targeting the MAPK pathway may be similarly governed by tissue architecture, we recreated an established tumor and drug regimen using a 3D soft agar assay, in which we administered drug treatment once every three days. This time frame was designed to establish tumors that lacked necrotic cores. Then we administered drug treatment that mimics clinical settings (Fig. 3 Top panel). Colonies derived from A375 cells were largely insensitive to cisplatin, vemurafenib, vx-11e and vemurafenib + vx-11e co-incubation. Contrary to A375 cells, the A375.S2 colonies showed increased sensitivity to drugs targeting the MAPK signaling pathway as determined by a reduction of number of colonies concomitant with reduced colony diameters (Fig. 3 Bottom Panel).

**Fig. 4:**
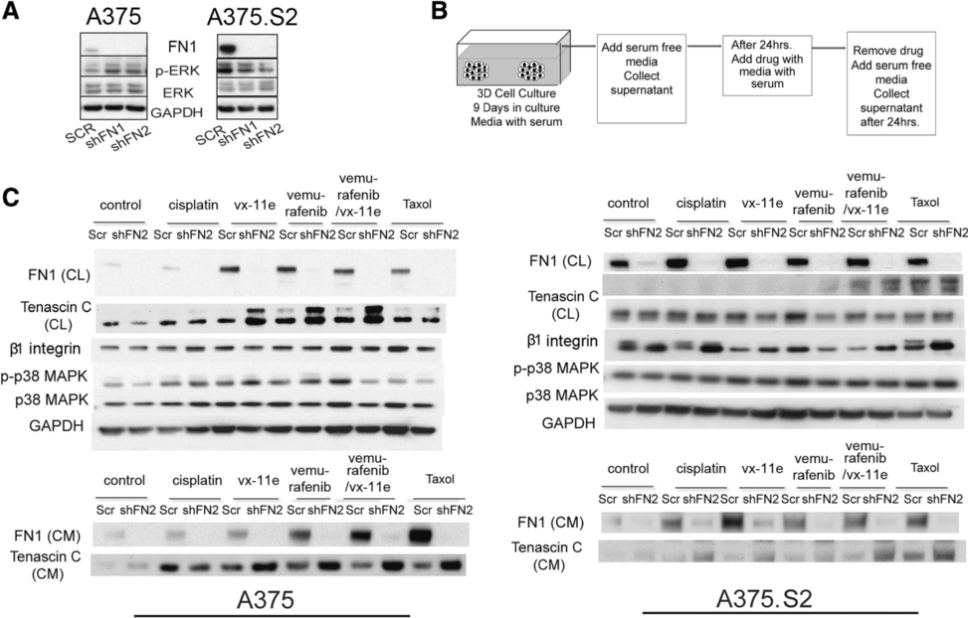
A375 tumor cells that survive pharmacological inhibition of MAPK signaling pathway upregulate FN. **a** Immunoblots show the reduction of FN levels using shRNA has a modest effect on ERK activity for two shRNA FN constructs (shFN1 and shFN2). **b** Schematic of drug treatment regimen: tumors were grown in 3D from single cells embedded in laminin-rich ECM (lrECM) for 9 days, replenished with serum-free media for 24 h, followed by drug treatment in serum-containing media, and replenished for 24 h with serum-free media for harvesting. **c** Immunoblots show differential response of isogenic clones to drug treatment. The A375 clone shows an increase in FN production, whereas the A375.S2 clone shows little in response to drug treatment. Immunoblots of conditioned media (CM) and cell lysates (CL) pre- and post-drug treatment show an increase in FN levels in response to vx-11e, vemurafenib and combined treatment with vemurafenib and vx-11e, but not to cisplatin. TNC was upregulated in the shFN2 aggregates in response to treatment. Equal amounts of protein from conditioned media were analyzed on separate gels for measuring FN and TNC levels

We reasoned that the ability to modulate FN secretion might mediate survival in the parental clone as compared to the A375.S2 cells. To determine if heterogeneity in FN deposition mediated the observed differential drug sensitivity between the clones, we generated two stable cell lines, named shFN1 and shFN2, producing reduced levels of FN using two shRNAs, respectively. In addition, a scramble control (Scr) was made. These three cell lines were cultured in 3D using soft agar for 21 days before protein extraction. Immunoblots revealed that an approximately 80 % reduction of FN protein was found in shFN1 and shFN2 cell lines, compared to the Scr cells. However, the shFN1 and shFN2 cells did not show significant difference in ERK activation (Fig. 4a). To determine if cell-ECM crosstalk was impaired with reduced FN biogenesis, we examined expression of integrins that facilitate cross talk with FN, and modulate cellular attachment to the ECM in vivo. Comparing the shFN1 and shFN2 cells, αv integrin expression was different but β1 integrin was not (Additional file 1: Figure S2). Besides, shFN1 and shFN2 cells expressed higher levels of β1 integrin than the wildtype and Scr counterparts, while FN knockdown did not affect αv integrin expression level (Additional file 1: Figure S2). This highlights that shFN cells maintain the machinery for cell-ECM communication, and suggests that β1 integrin levels correlate with FN modulation. Similarly, we asked if altered cell cycle progression may account for differential sensitivity. We determined that the cell cycle was unchanged in shFN cells compared to Scr cells (Additional file 1: Figure S3).

### FN deficiency increases Tenascin-C

We next examined the secretome profiles of cells cultured in 3D lrECM. Culture medium from A375 and A375.S2 cells were collected before and after drug treatment (Fig. 4b). In A375 Scr cells, FN was found to increase at the cellular level in response to vx-11e, vemurafenib and paclitaxel treatments (Fig. 4c- Left panel). The surviving population tuned FN biogenesis under stress, particularly noting the dramatic increase following BRAF and ERK inhibition, but not for cisplatin and paclitaxel. FN-deficient aggregates, on the other hand, showed upregulation of Tenascin-C (TNC) in response to drug treatment. In contrast, the A375.S2 cells showed only a modest response despite displaying higher baseline levels of FN, which correlates with the reduced vitality of A375.S2 colonies cultured in agar (Fig. 4c- Right panel).

### FN reduction sensitizes melanoma colonies to cisplatin, vemurafenib and Vx-11e

**Fig. 5:**
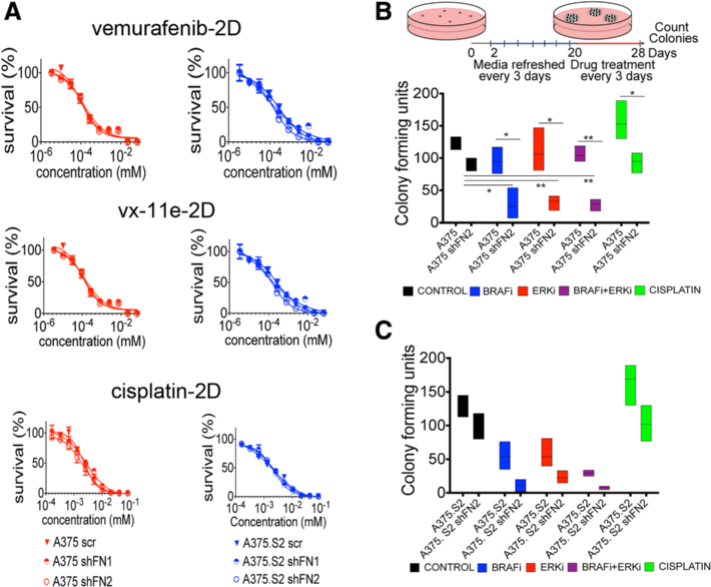
Reduced FN render previously insensitive, 3D tumor aggregates susceptible to a broad range of drugs. **a** Viability curves of Scr and shFN cells treated with MTT in 2D show negligible differences for pharmacological inhibitors cisplatin, vemurafenib and vx-11e. **b** Schematic showing the timing of drug administration for cells embedded in 3D soft agar with treatment commencing after colony formation. A375-Scr cells are insensitive to inhibitors of the MAPK pathway, whereas A375 shFN2 cells show increased sensitivity to MAPK inhibition at dosages determined from the IC_50_ curves. **c** Similar comparison of colonies formed from A375. S2-Scr cells show greater sensitivity to inhibitors of the MAPK pathway. Colonies of A375.S2 shFN2 cells were further reduced in number following treatment by all inhibitors. Significance was determined by student’s *t*- test, comparing within conditions to control where * indicates *p* <0.05, ** indicates *p* <0.01, *** indicates *p* <0.001, **** indicates a *p* <0.0001

To determine whether FN reduction in melanoma cells could affect drug sensitivity, we assessed the sensitivity of the A375, A375.S2, with their Scr and shFN clones, with vemurafenib, vx-11e and cisplatin cultured in 2D and 3D environments. The half maximal inhibitory concentration (IC_50_), as determined from the viability curves constructed post-MTT assay in 2D, remains unchanged despite the reduced levels of FN (Fig. 5a). By measuring cellular uptake of nitrotetrazolium blue chloride, we confirmed that the cells were metabolically active (Additional file 1: Figure S4B). However, we confirmed changes in intracellular signaling in response to treatment by assessing ERK activity in addition to MAPK activation (Additional file 1: Figure S4A), suggesting that observed sensitivity of FN-deficient colonies correlates with molecular events tied to 3D architecture establishment. Both Scr and shFN cell lines responded to treatment when BRAF^V600E^ and ERK inhibitors were administered before colony formation (Additional file 1: Figure S4C). Interestingly, normal resistance to cisplatin observed in wild type malignant melanoma cells was diminished in the shFN cell lines in this assay. When cells were allowed to form colonies, those colonies derived from control Scr cells remained largely intact and insensitive to any of the drugs administered (Fig. 5b). shFN colonies were sensitive, showing a reduced number of surviving colonies following drug treatment (Fig. 5b). FN-depleted A375.S2 colonies showed an exaggerated sensitivity to drugs targeting MAPK signaling (Fig. 5c).

### FN expression is part of signaling pathway in drug response

**Fig. 6:**
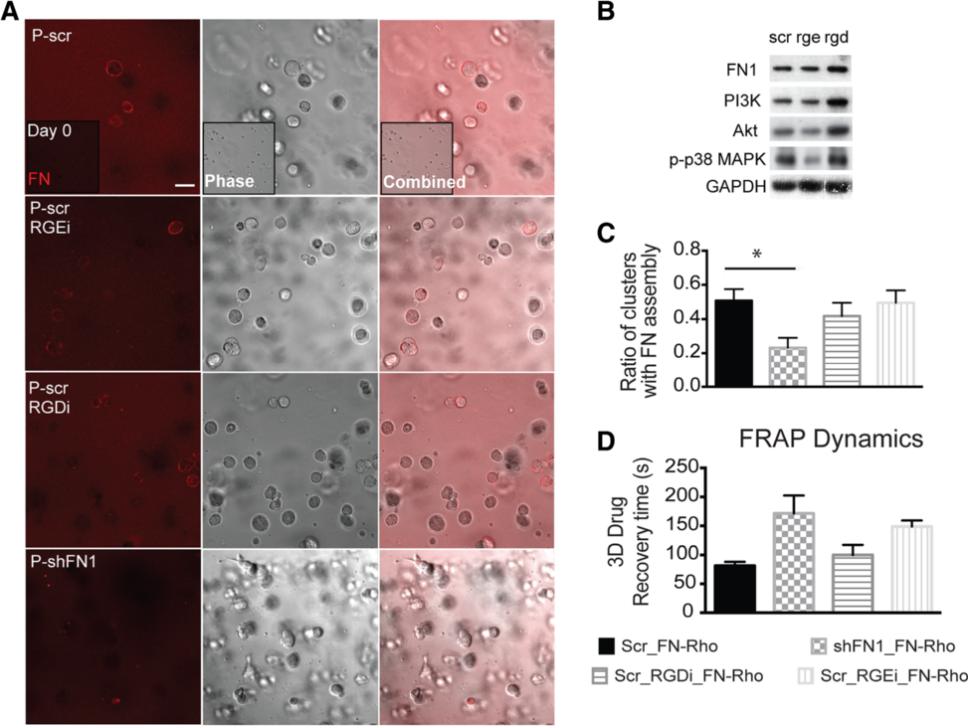
FN assembly is impaired in shFN1 cells cultured in 3D lrECM **a** Representative images of aggregates 2 days after exogenous FN conjugated to Rhodamine (Rho) is added to 3D lrECM culture (inset shows no FN assembly on day 0). IgG control and integrin-blocking peptides were compared in shFN and Scr cells. Scale bar represents 20 μm. **b** Immunoblots show that FN and phospho-p38, but not other proliferation markers in the presence of blocking peptides. **c** Graph show a reduction in the number of shFN2 colonies that assemble exogenous FN as a cortical ring compared to Scr colonies **d** Drug uptake is only modestly slower as determined from fluorescent recovery after photobleaching. Graph showing that the recovery time for drug influx is only modestly slower for shFN2 3D aggregates. Conditioned media to probe FN and TNC was run on separate gels where equally loaded protein

To understand the role of FN architecture in determining malignant melanoma tumor sensitivity to treatment, we sought to determine whether drug uptake was endogenously affected in shFN cells, or if assembly of bioavailable fibronectin would rescue the observed sensitivity. First, we added exogenous fluorescent-labeled FN to Scr and shFN cells that were cultured in 3D lrECM. As a further control, the Scr cell line was incubated with and without RGD motif-blocking peptides. We observed assembly of FN into *de novo* cortical rings formed 48 h post-addition by control cells (Fig. 6a). Immunoblots revealed that FN levels remained constant despite the presence of blocking peptides (Fig. 6b). However, fewer shFN aggregates assembled cortical FN (Fig. 6c). Using fluorescent conjugated taxol, we found that drug uptake in 2D culture was unaffected (Additional file 1: Figure S5), but fluorescence recovery after photobleaching (FRAP) in shFN 3D aggregates revealed that drug recovery time was modestly slower (Fig. 6d).

**Fig. 7:**
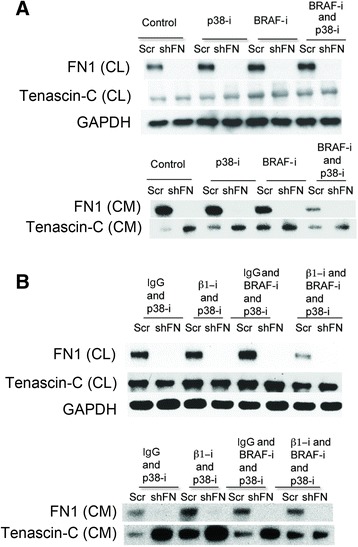
FN assembly and Tenascin-C are mediated by p38 MAPK and β1 integrin. **a** Immunoblots reveal no change in FN levels (cell lysate and conditioned media) when p38 MAPK is inhibited. **b** Immunoblots reveal that β1 integrin modestly affects FN secretion, but significantly increases TNC in both cell lines (Scr and shFN2)

These data suggested in addition to providing a physical barrier, secreted FN might be part of an essential signaling cascade in responds to extracellular stress. We observed that p38 signaling was increased after drug treatment (Fig. 4c). Combined treatment with BRAF^V600E^ and p38 MAPK inhibitors consequently reduced FN secretion in Scr cells, and lowered TNC expression in shFN cells (Fig. 7a). This suggests that p38 MAPK activity is necessary to fully upregulate FN (or TNC if FN production is impaired) in response to drug. Earlier we suggested a role for β1 integrin in similarly modulating FN biogenesis. Cells grown in the presence of β1 integrin-blocking antibody, BRAF and p38 inhibitors produce drastically less FN (Fig. 7b). p38 MAPK and β1 integrin inhibition prevented malignant melanoma cells from modulating FN expression, which was shown to be essential in surviving pharmaceutical stress.

## Discussion

Treatment strategies for metastatic malignant melanoma have significantly improved due to our current understanding of the molecular basis of tumor etiology [29]. Targeted therapy based on BRAF V600E status has yielded dramatic results and disease regression has been observed. In spite of recent progress, the stark reality is that current treatments do not yet achieve durable responses due to melanoma intratumoral heterogeneity. The remodeling of the ECM results in changes within the microenvironment adding further complexity to the determinants of drug efficacy and the likelihood of acquired resistance. We present a novel mechanism by which cells tune their ECM secretion, namely FN via p38 MAPK –β1 integrin, in response to pharmaceutical inhibition of the MAPK signaling pathway in cutaneous melanoma cell lines, especially in 3D culture system. Here we show that when FN biogenesis is impaired, increased TNC expression is observed, suggesting a compensatory mechanism to attenuate drug treatment. By using the A375 cell lines that mimic tumor heterogeneity, we determined that only clones that can modulate FN biogenesis survive pharmaceutical intervention. These results, in conjunction with similar observations in patient data, suggest a role for ECM biosynthesis in the emergence of *de novo* resistance.

Extracellular matrix proteins (ECMs) are often observed to be abundantly expressed by tumor cells in comparison to their normal counterparts. Analysis of patient data confirmed that overexpression of ECM proteins such as FN and TNC are likewise observed in patients that had acquired *de novo* resistance from initial drug therapies. Similar outcomes have been noted for breast, laryngeal, urothelial and non-small-cell lung cancers [22, 30–32]. Using proteomics and immunofluorescence, we determined a distinct temporal distribution of secreted ECM components and visualized the assembled ECM scaffold for two isogenic malignant melanoma clones. We determined that melanoma cells modulate the secretion of many ECM proteins that are also implicated as potential biomarkers for patients suffering from metastatic disease [33]. Recently, ECM signatures were assigned to breast and ovarian tumors of differing metastatic potential [34, 35]. These ECM signatures were not solely derived from the stromal cell secretions, but also included contributions from the tumor cells themselves [35]. Here, we go a step further to follow the evolution of a melanoma-derived ECM signature. In particular, the secretion patterns of the two clones were significantly different with respect to relative amounts of FN, periostin, TNC, laminins and vitronectin (Fig. 1c). FN also differed architecturally; fibrils were predominantly observed in one clone vs. globular scaffolds for the other (Fig. 2d). FN fibril assembly is regulated by tensional reorganization of actin via stress fibers for cells cultured in 2D [36–38]. Actin structures convey different mechanical properties based on spatial organization in 2D, and regulate specific motilities in 3D [25, 39]. Furthermore, cell motility regulates ECM remodeling and plays a role in the final architecture of the ECM scaffold [40]. These two cell lines show differential adhesion forces when placed in a 2D biomimetic platform simulating cell-ECM interactions [41]. Additionally, differences in FN conformation dictate the availability of binding sites that affect signaling cascades associated with growth, migration and adhesion [42, 43]. In our system, it remains to be seen if there is a difference in tensional regulation and whether globular vs. fibril conformations show differential drug sensitivity.

In this study, we observed that FN was differentially secreted and assembled when tumor cells harboring the BRAF^V600E^ mutation were cultured in 2D vs. in a biomimetic 3D platform [44]. In vivo, tumor cells have access to various sources of fibronectin, namely the plasma and stroma. As a surrogate basement membrane gel, the laminin-rich ECM contains several ECM proteins and cytokines, including minute amounts of FN [40], However, previous studies have shown that FN-null cells poorly assemble FN fibrils when exogenous FN is added to them [45]. We confirmed that a reduced number of shFN cells assembled exogenous FN compared to Scr cells in in vivo and in vitro 3D culture (Figs. 3 and 7). Growth inhibition when FN has been compromised has also been observed for other types of cancer [46]. This was due in part to reduced expression of β1 integrin and alterations of the cell cycle; however, in our system the short hairpin did not alter the cell cycle or reduce β1 integrin levels. Melanoma-derived conditioned media was shown to stimulate in vivo FN expression in target organs preceding future metastatic lesions. This phenomenon was also attributed to resident fibroblasts that create the “pre-metastatic niche” [47]. Our data suggests that metastatic melanoma also modulates its “tumor niche” by secreting and assembling its own ECM scaffold to survive drug treatment. Our results suggest that modulation of FN secretion serves as a response to external stress and may be separate from the remodeling of stromal components observed during initial malignant transformation.

Melanoma is largely resistant to platinum treatments since several factors such as drug uptake, increased efflux, extracellular interaction via cell adhesions and slow cycling cells may contribute to intrinsic resistance [48–50]. In our system 3D architecture did mitigate drug efficacy, as only a partial killing was achieved for FN-null tumor aggregates and minimal FN secretion was observed in response to cisplatin.

It has been previously reported that activation of the ERK/MAP kinase in melanoma cells stimulates FN biogenesis via the early growth response-1 transcription factor (Egr-1) [51]. High FN and Egr-1 levels were found in BRAF^V600E^ mutant cells and patient tumors. However, stimulation of the ERK pathway was insufficient to induce FN biogenesis in normal melanocytes [51]. Here, inhibition of the ERK pathway activates FN biogenesis via p38MAPK and β1 integrin signaling in melanoma cells but not in melanocytes. In a previous study, β1 integrin inhibition targeting FN has increased radio sensitization of breast tumor cells in 3D culture [52]. However, non-malignant breast cells undergo apoptosis following treatment with a β1-integrin function-blocking antibody, whereas the malignant counterpart cells appear resistant to this treatment [53, 54]. Integrin blocking treatment is successful only if there is a differential response between normal tissue and its malignant counterpart. Thus, in addition to multiple targets of MAPK signaling, concomitant treatment with a p38 inhibitor may be a better strategy to prevent acquire drug resistance due to FN biosynthesis. However, in the case of metastatic disease, acute treatment with integrin-blocking antibodies may be useful to overcome *de novo* resistance. Nevertheless, alternative strategies to overcome FN mediated cell adhesion mediated drug resistance have been reported that could reverse drug resistance in leukemia and solid tumors [55, 56].

In normal cells during early embryogenesis, FN provides a scaffold for the assembly of other ECM proteins to direct organ specificity and fate, so we reasoned that other ECM proteins would be altered when FN biogenesis is affected [57]. In fact, we observed an increase in Tenascin-C biogenesis by shFN melanoma cells. Here, we show that drug treatment may induce ECM biosynthesis, contributing to *de novo* resistance. This must be considered when designing therapeutic interventions. Our findings suggest that modulation of tumor ECM biogenesis mimics the behavior of bacteria that assemble a biofilm in response to nutrient deficiencies, antibiotics and oxidative stress [58]. This biofilm acts as a physical barrier and signaling reservoir to communicate sensing of deleterious conditions. Tumor cells may use this secreted “biofilm” to communicate and communally enter a state of dormancy until environmental conditions are more favorable. If we can understand the mechanisms underlying tumor biofilm assembly, we may be able to seize a novel opportunity for therapeutic intervention.

## Conclusions

In this study, we observed an increase in Tenascin-C biogenesis in melanoma cells expressing reduced level of fibronectin, suggesting a compensatory mechanism in response to drug treatment. We also showed that drug treatment could induce ECM biosynthesis, contributing to *de novo* resistance. Our findings suggest that modulation of tumor ECM biogenesis mimics the behavior of bacteria that assemble a biofilm in response to nutrient deficiencies, antibiotics and oxidative stress.

### Ethics approval and consent to participate

The data shown in Fig. 2b was generated by using publicly available RNAseq data from human patients. No ethics approval and consent to participate documents are necessary.

### Consent to publish

This manuscript does not contain data from any individual person’s data in any form require consent to publish from that person, or in the case of children, their parent or legal guardian.

### Availability of data and materials

The datasets supporting the conclusions of this article are included within the article (and its additional files).

## Additional file

Additional file 1:
**Supplemental Figures.** (PDF 1475 kb)

## Abbreviations

ECM: extracellular matrix
FN: fibronectin
lrECM: laminin-rich ECM
MAPK: mitogen-activated protein kinase
TNC: tenascin-C
