# Supplementary material for: Mitogen-activated protein kinase signaling causes malignant melanoma cells to differentially alter extracellular matrix biosynthesis to promote cell survival
Source: BMC Cancer. 2016 Mar 5;16:186. doi: 10.1186/s12885-016-2211-7 (PMC4779217; doi:10.1186/s12885-016-2211-7)
Supplement: Additional file 1: — Supplemental Figures. (PDF 1475 kb) [file 12885_2016_2211_MOESM1_ESM.pdf]

Supplemental Figures

Malignant Melanoma Cells Differentially Alter Extracellular Matrix Biosynthesis that Promotes Survival in Response to Targets of Mitogen-Activated Protein Kinases Signaling

Anna Afasizheva[1#], Alexus Devine[1#], Heather Tillman[2], King Leung Fung[1], Benjamin H Blehm[1], Yorihisa Kotobuki[1], Ben Busby[3], Emily I Chen[4], Kandice Tanner[1*]

[1]Laboratory of Cell Biology, Center for Cancer Research, National Cancer Institute, National Institutes of Health
[2]Laboratories of Genitourinary Cancer Pathogenesis, Center for Cancer Research, National Cancer Institute, National Institutes of Health
[3] National Centers for Biotechnology Information, National Library of Medicine, NIH
[4] Proteomics Shared Resource at the Herbert Irving Comprehensive Cancer Center & Department of Pharmacology, Columbia University Medical Center

[*] To whom correspondence should be addressed: Kandice Tanner Ph.D., 37 Convent Dr., Bethesda, MD 20892
kandice.tanner@nih.gov

#Authors contributed equally

Supplemental Figure 1. Examination of integrins that mediate cell ECM interactions

A) Top panel- Schematic of 3D sample preparation. Cells were embedded in laminin rich ECM (lrECM) and cultured for 10 days to recapitulate growth of melanoma in the presence of basement membrane.

(B) Quantification of tumor aggregates shows differences in size and shape (circularity) between aggregates derived from distinct clones, where A375 aggregates are bigger and irregular while A375.S2 clones are smaller and circular. *** indicates a p value<0.001

(C) Real-time PCR confirm that both clones produce FN1 by probing mRNA levels. Actin is a loading control.

Supplemental Figure 2. Examination of integrins that mediate cell ECM interactions

Immunoblots show that αv integrin levels are similar for Scr control cells and shFN cells, whereas β1 levels show a modest increase in shFN cells. GAPDH is a loading control.

Supplemental Figure 3. Cell cycle is unaltered for shFN cells compared to Scr control Tumor cells

Cell cycle analysis revealed negligible differences between shFN cells and scramble control for both isogenic melanoma clones.

Supplemental Figure 4. Effect of architecture on drug response

A) Immunoblots of protein cultured in 2D show differential ERK phosphorylation for each clone in response to inhibitors, despite previously established similarity of viability curves.

(B) Representative images show that colonies are viable and metabolically active as determined by uptake of nitrotetrazolium blue chloride.

(C) Histograms showing the size distribution and numbers of colonies after one month, where drug treatment commenced at the single cell stage in 3D soft agar.

Supplemental Figure 5. 2D drug uptake is unaltered for shFN cells compared to Scr control Tumor cells

Control studies show that rhodamine administered with an inhibitor of a drug transporter illustrates no differences in uptake observed between Scr and shFN cells in 2D (Left panel). Similarly, no differences were observed for fluorescent conjugated taxol (right panel).

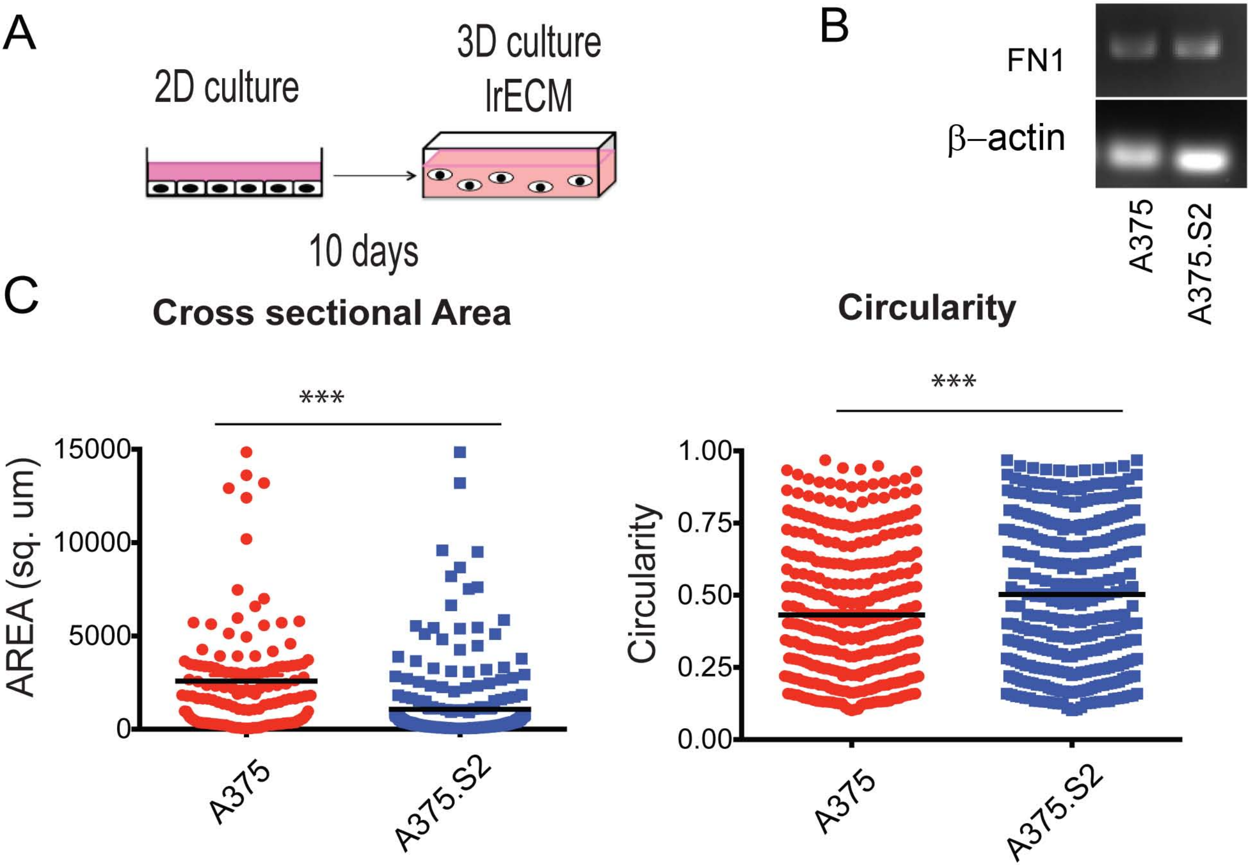

Supplemental Figure 1

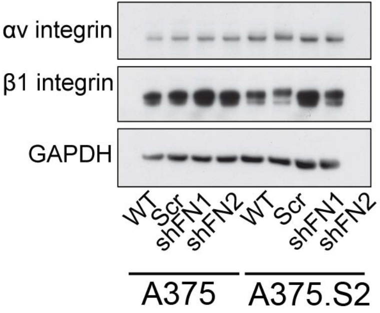

Supplemental Figure 2

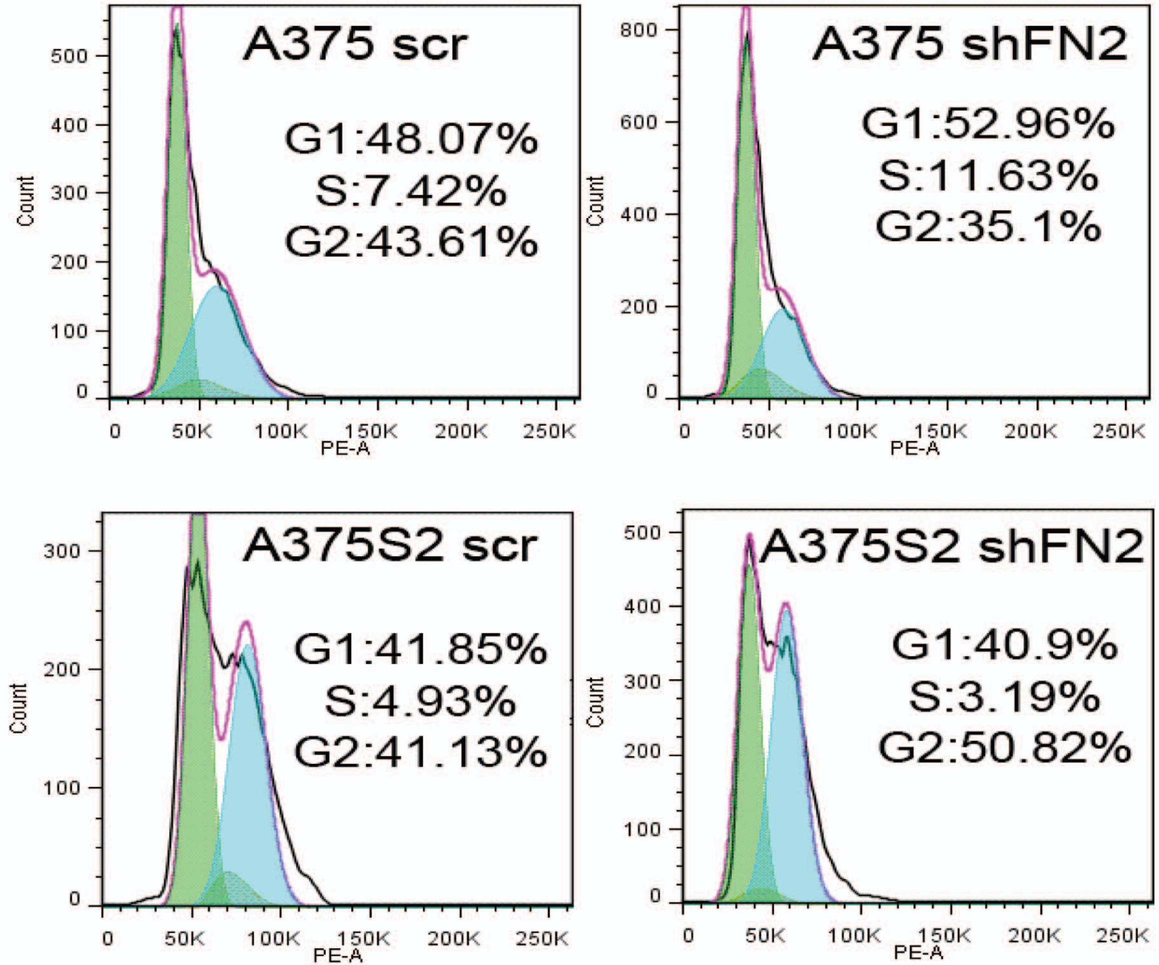

Supplemental Figure 3

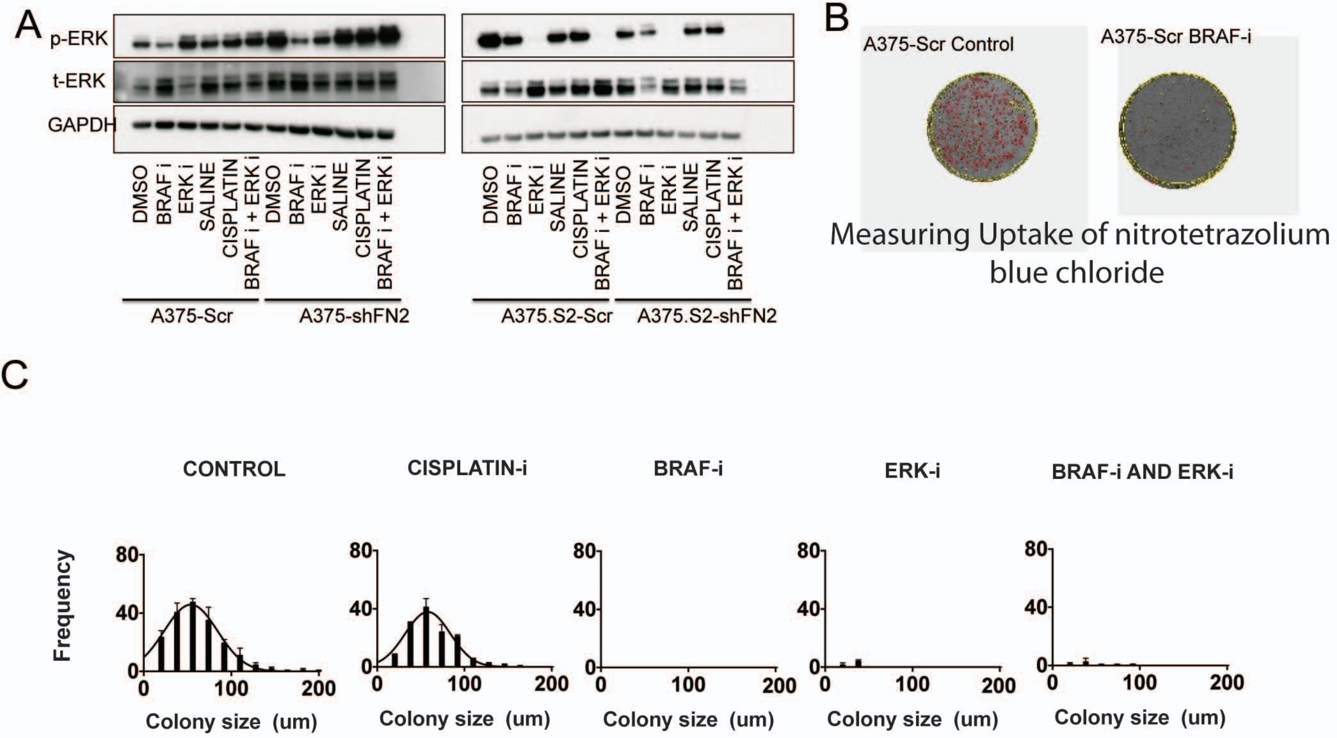

Supplemental Figure 4

A)

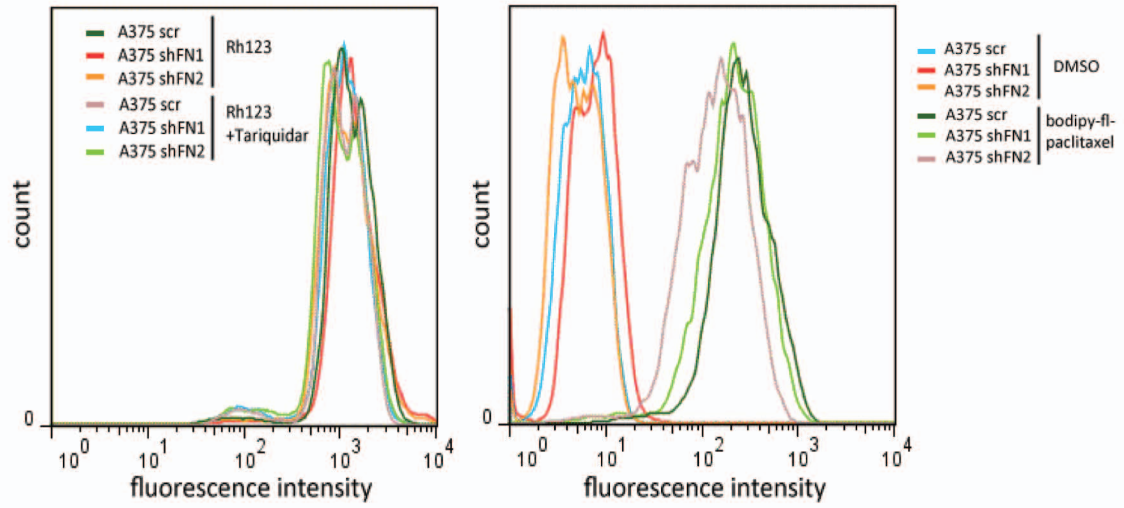

Supplemental Figure 5
